# A Rare Case of Primary Anterior Mediastinal Yolk Sac Tumor in an Elderly Adult Male

**DOI:** 10.1155/2016/8961486

**Published:** 2016-04-06

**Authors:** Sammy G. Nakhla, Srinath Sundararajan

**Affiliations:** ^1^Department of Medicine, Southern Arizona VA Health Care System, Tucson, AZ 85723, USA; ^2^Department of Hematology/Oncology, University of Arizona Cancer Center, Tucson, AZ 85724, USA

## Abstract

Mediastinal germ cell tumors are extragonadal germ cell tumors (EGGCTs) commonly seen in children and young adults. They are more common in men. Clinically they are classified as teratomas, seminomas, and nonseminomatous germ cell tumors. Primary mediastinal yolk sac neoplasm is an extremely rare tumor. We present here a very rare case of primary yolk sac tumor of the anterior mediastinum in a 73-year-old male. Mediastinal germ cell tumors have a worse prognosis than gonadal germ cell tumors. Chemotherapy followed by adjuvant surgery improves overall response in EGGCTs. However, comorbidities can render treatment with chemotherapy and surgery challenging in elderly patients.

## 1. Introduction

Yolk sac tumor is a subtype of germ cell tumor that is highly malignant. In addition to presenting in ovaries and testes, the tumor has been detected at several extragonadal sites such as the retroperitoneum, sacrococcygeal region, pineal gland, and the anterior mediastinum [1]. Primary yolk sac tumor (YST) of the anterior mediastinum is rare and has a grave prognosis. Often patients present with advanced stage tumors that are bulky and unresectable [2]. Like other germ cell tumors (GCTs), YST is predominantly a disease of young adults and the average age at diagnosis is 18 years [1]. However, a few cases of gonadal and extragonadal germ cell tumors have been reported in elderly patients as well [3–6]. We present a very rare case of an elderly 73-year-old male with primary yolk sac tumor of the mediastinum.

## 2. Case Report

**Figure 1:**
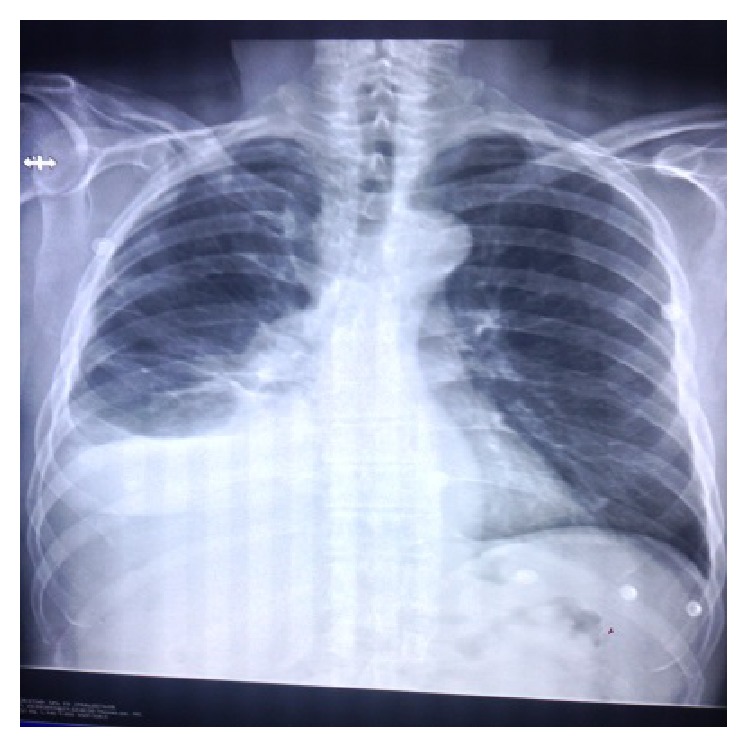
Chest X-ray of 73-year-old male revealing large right unilateral pleural effusion.

**Figure 2:**
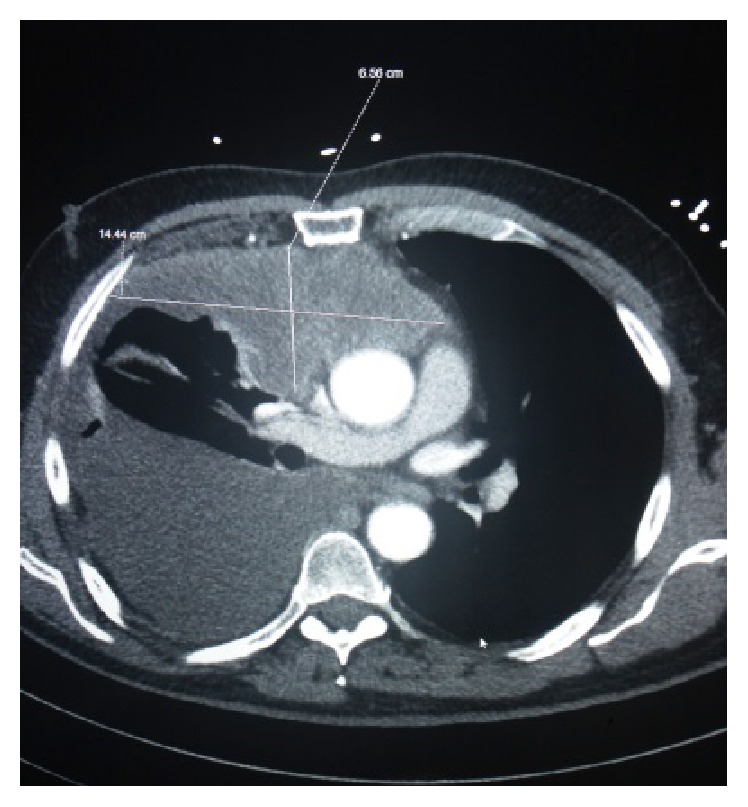
Computerized tomography (CT) scans of chest revealed a large mass/conglomerate lymphadenopathy measuring 6.6 cm × 14.5 cm × 7 cm in the anterior inferior mediastinum extending to involve the anterior right hemithorax, along the pleural surface, associated with a very large right-sided pleural effusion, causing partial collapse of the right inferior and middle lobes.

A 73-year-old Caucasian male with past medical history of chronic lymphocytic leukemia, atrial fibrillation, and dyslipidemia presented to the emergency department with complaints of worsening dyspnea on exertion for approximately three to four days and occasional night sweat. He did not have fever, chills, night sweat, cough, sputum production, sick contacts, or recent travel. His physical examination was unrevealing except for right mid posterior egophony and decreased breath sounds in the right lung base. His labs revealed a leukocytosis of 15 × 10^3^ cells/uL with 70% lymphocytes and smudge cells present consistent with his prior CLL. Patient's chest X-ray revealed a large right unilateral pleural effusion with loculation. A computerized tomography (CT) scans of his chest, abdomen, and pelvis revealed a large mass/conglomerate lymphadenopathy measuring 6.6 cm × 14.5 cm × 7 cm in the anterior inferior mediastinum extending to involve the anterior right hemithorax, along the pleural surface, associated with a very large right-sided pleural effusion, causing partial collapse of the right inferior and middle lobes (Figures 1 and 2).

**Figure 3:**
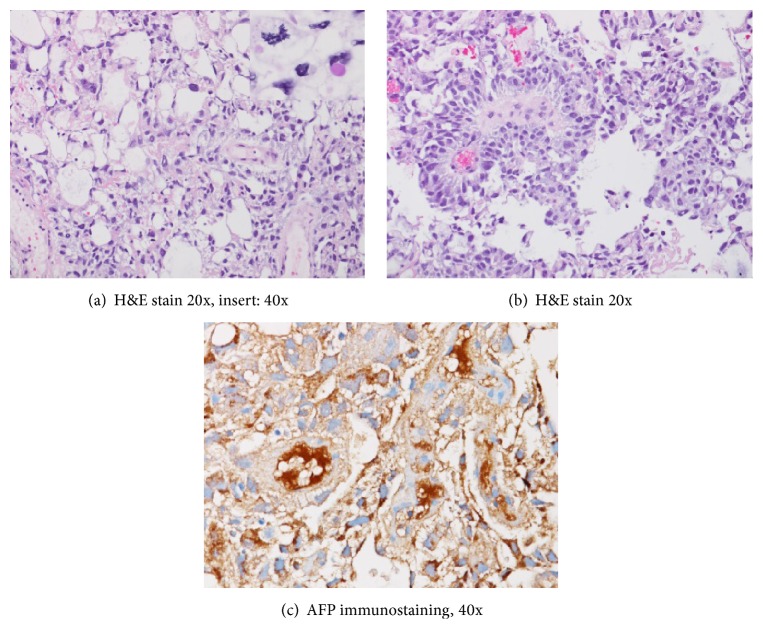
The fine needle aspiration/core biopsy revealed abundant tumor cells that were pleomorphic with reticular growth pattern. The tumor showed focal reactivity with AFP and CDX2 and focal weak reactivity with TTF-1 and is nonreactive with CK7, CK20, Napsin A, p63, calretinin, CK5/6, D2-40, CD31, PSA, PSAP, synaptophysin, chromogranin, CD56, hepar, CD45, CD5, CD10, and CD30.

The patient was worked up for an anterior mediastinal mass. Tests for alpha-fetoprotein (AFP), LDH, beta-human chorionic gonadotropin (B-HCG), thyroid-stimulating hormone, and cortisol were requested. AFP was elevated at 5,610 IU/ML, serum beta-HCG < 2.0 mIU/mL, TSH was at 2.11 mIU/L, and LDH was 903 U/L. Ultrasound examination of the scrotum was done as well, results of which were negative. Fine needle aspiration of the mediastinal mass revealed abundant tumor cells that were pleomorphic with reticular growth pattern (Figures 3(a), 3(b), and 3(c)). Immunohistochemistry showed focal reactivity with AFP and CDX2 and focal weak reactivity with TTF-1. The tumor cells were nonreactive with CK7, CK20, Napsin A, p63, calretinin, CK5/6, D2-40, CD31, PSA, PSAP, synaptophysin, chromogranin, CD56, hepar, CD45, CD5, CD10, and CD30. Based on the light microscopy morphology and immune-phenotype, final diagnosis of a yolk sac tumor, pleomorphic variant was made. Considering the negative ultrasound of the scrotum, patient was diagnosed to have an extragonadal or primary anterior mediastinal yolk sac tumor (YST). Patient was referred to oncology and had a PleurX chest catheter placed for drainage of right-sided effusion prior to discharge.

Patient was readmitted within a few days with worsening shortness of breath and new segmental pulmonary embolism. He was started on anticoagulation and was treated as inpatient with chemotherapy. The patient was treated with VIP regimen comprising of etoposide (Vepesid) at 80 mg/m^2^, ifosfamide at 1200 mg/m^2^ (with mesna 120 mg/m^2^), and cisplatin 20 mg/m^2^ on days 1–5. Granulocyte colony stimulating factor was started prophylactically starting day 7. His postchemotherapy course was complicated by pancytopenia, hemothorax, sepsis, and multiorgan failure. Due to continued decline of his overall health status despite aggressive supportive measures, patient's family resorted to comfort care measures and all life support measures were withdrawn.

## 3. Discussion

Germ cell tumors (GCTs) mostly occur in the gonads. Extragonadal germ cell tumors (ECGCTs) are rare and most can arise in the pineal gland, retroperitoneum, and the mediastinum [7]. EGGCTs have been speculated to develop from primordial germ cell remnants that fail to migrate to gonadal ridge during embryogenesis [2]. Hence, the locations of development of EGGCT fall along the migration pathway of the primordial germ cells to gonadal ridge. The mediastinum is the most common site of EGGCTs [8]. Malignant GCT in the mediastinum accounts for 1–6% of all mediastinal tumors [9]. Primary extragonadal GCTs, especially primary mediastinal tumors, are considered to have poor prognosis [10].

GCTs are histologically categorized into teratomas, teratocarcinomas, seminomas, and nonseminomatous carcinoma, including choriocarcinoma, embryonal carcinoma, yolk sac carcinoma, and mixed type carcinoma [1]. Greater than 90% of malignant extragonadal tumors of the mediastinum occur in men. In a retrospective study by Sakurai et al. with 48 patients, the median age at presentation of extragonadal GCT was 28.8 years [11].

Yolk sac tumors (YSTs) can occur in both men and women, usually arising from germ cells in testes and ovaries, respectively. Pure YST tumors are usually found in young children and mixed germ cell tumors with YST are found in adults [1]. Yolk sac tumors, similar to other nonseminomatous germ tumors, can be associated with hematologic Klinefelter's syndrome (up to 20%) and other hematological malignancies such as acute leukemia and myelodysplastic syndrome [12]. In an international study by Bokemeyer et al. with 381 mediastinal GCTs, the most common symptoms on presentation were dyspnea (25%), chest pain (23%), cough (17%), fever (13%), night sweat, or weight loss (11%). Night sweat, fatigue, hemoptysis, and symptoms of superior vena cava compression were seen in <10% of patients with mediastinal GCT [7].

Histologically, extragonadal GCTs and mediastinal GCTs have many similarities. Microcystic/reticular pattern is the most common histological presentation [13]. Schiller-Duval bodies are pathognomonic and are helpful for identification. Yolk sac tumors immunohistochemical testing is positive for AFP, glypican-3, SALL4, and placental alkaline phosphatase. In a study by Moran et al. with 38 YST patients, the most common pattern observed was the reticular type, characterized by strands and cords of cells embedded in a myxoid or edematous stroma [12]. Yolk sac tumors secrete substances such as AFP, B-hCG, and cytokeratin (CEA), which can be measured in the serum [10]. Although extragonadal GCTs and mediastinal GCTs have similar histologic feature, they are clinically and biologically distinct from their testicular counterparts [11].

The treatment regimens of extragonadal and gonadal YSTs are similar since they share histological patterns. Extragonadal nonseminomatous germ cell tumors have considerably poorer prognosis. Chemotherapeutic schemes based on cisplatin have shown significant results with up to 50% of patients achieving long-term survival [14, 15]. Bleomycin-Etoposide-Cisplatin (BEP) therapy or etoposide (Vepesid), ifosfamide, and cisplatin (VIP), at least 4 cycles, are widely accepted chemotherapy regimens [16, 17]. VIP regimen may be preferred over BEP since patients with mediastinal GCTs might need postchemotherapy thoracotomy for removal of residual tumor and bleomycin induced pulmonary toxicities can be potentiated by surgery.

Surgical resection as the primary treatment modality is not recommended in mediastinal GCTs because of the likelihood of early metastasis [11]. However, there is a definite role for postchemotherapy adjuvant surgery to remove residual lesions and a rising serum tumor marker after completion of chemotherapy is not considered as a contraindication for surgery [18, 19]. Complete resolution of serum AFP marker occurs in less than 5% of patients [20]. Survival rates have increased in patients who have decrease in serum of AFP after chemotherapy and in cases with residual tumor surgical excision [21]. According to the International Germ Cell Collaborative Group, prognosis is considered poor (5-year survival rate of 48%), if only one of three following criteria is met: primary mediastinal locations of the NSGCT, the nonpulmonary visceral metastases, and an AFP level greater than 10,000 IU/mL [22].

Elderly patients with germ cell tumor generally have worse clinical outcomes compared to younger patients. A Surveillance, Epidemiology, and End Results (SEER) database analysis with 12,811 patients compared the outcomes of testicular cancers in young adults (age < 50) versus older adults (age > 50 years) [23]. In this study, survival from both localized and metastatic NSGCT was noted to be much better in younger patients compared to elderly (76.9% versus 57.0%). Another study that evaluated the clinic-pathological features and tolerance of platinum based chemotherapy of 50 older adults (age > 50) treated for testicular germ cell tumor noted that 30 of 50 (60%) patients had significant dose delays or could not complete their planned chemotherapy course and a majority of patients required regimen change [6]. Neutropenic fever was noted in 44% of patients overall and in 12% of patients despite prophylactic G-CSF. Our patient had overall poor prognosis due to multiple reasons such as his age, tumor location, and tumor bulk.

## 4. Conclusion

Extragonadal and mediastinal GCTs are diseases of childhood or early adulthood. It rarely appears after the age of 30 years. Primary mediastinal yolk sac neoplasm is a rare tumor. To the best of our knowledge, this is the first reported case of a primary yolk sac tumor of the mediastinum in an elderly 73-year-old male. High index of suspicion is needed to diagnose extragonadal GCTs in elderly patients. Diagnosis can be made with elevated AFP in conjunction with a supporting histopathology. Elderly patients with EGGCT have considerably worse outcome due to their functional status and comorbidities.
